# Hodgkin Variant Richter's Transformation in the Absence of Classical Risk Factors: A Rare Case With Spinal Manifestation

**DOI:** 10.7759/cureus.103575

**Published:** 2026-02-14

**Authors:** Evelin Kiss, Árpád Illés, Róbert Szász, Anna Rebeka Kovács, Gábor Méhes, Boglárka Brugós

**Affiliations:** 1 Faculty of Medicine, Department of Internal Medicine, Division of Hematology, University of Debrecen, Debrecen, HUN; 2 Medical Imaging Clinic, Department of Nuclear Medicine, Scanomed Ltd., University of Debrecen, Debrecen, HUN; 3 Faculty of Medicine, Department of Pathology, University of Debrecen, Debrecen, HUN

**Keywords:** brentuximab, cll, extranodal manifestation, hodgkin lymphoma, spinal cord

## Abstract

Richter’s transformation (RT) is a feared and not completely understood complication of chronic lymphocytic leukemia or small lymphocytic lymphoma (CLL/SLL). While most cases involve transformation to diffuse large B-cell lymphoma (DLBCL), CLL may, albeit rarely, progress to Hodgkin lymphoma (HL).

We report the case of a 74-year-old woman initially diagnosed with CLL/SLL who progressed to a rare form of HL subtype affecting the spinal cord. After receiving six cycles of brentuximab+doxorubicin, vinblastine, and dacarbazine (A+AVD) therapy at our Department of Hematology (University of Debrecen), the patient achieved complete metabolic remission (CMR) and remains in good condition.

HL-RT in CLL is relatively rare and generally associated with poorer outcomes, though it is typically more favorable than DLBCL-RT. In this case report, we highlight not only an uncommon anatomical location of HL-RT but also the absence of typical predisposing factors, such as a TP53 mutation, unmutated immunoglobulin heavy chain (IGHV) status, or a lack of 13q deletion.

## Introduction

Richter's syndrome (RS) or Richter’s transformation (RT) is a rare clinicopathological phenomenon that occurs in approximately 2% to 8% of patients with chronic lymphocytic leukemia or small lymphocytic lymphoma (CLL/SLL). The transformation refers to the progression of low-grade B-cell lymphoproliferative neoplasm into an aggressive lymphoma. Diffuse large B-cell lymphoma (DLBCL-RT) accounts for the majority (~90%) of RTs, whereas the Hodgkin-lymphoma variant (HL-RT) represents 0.7-15% of cases [1,2]. In more than 50% of cases, the molecular pathogenesis of RT includes a 17p deletion and/or a TP53 gene mutation, or genetic lesions affecting the NOTCH1 and MYC oncogenes. In contrast, 13q deletion and mutated immunoglobulin heavy chain (IGHV) status are considered protective against RT. In HL-RT, prognostic factors are less well-defined than in DLBCL-RT [3,4]. Histopathologically, the HL variant is characterized by Reed-Sternberg cells that are CD30- and CD15-positive and CD20-negative. Unlike DLBCL-RT, clonally, HL-RT cases are typically unrelated to CLL. The majority of patients are Epstein-Barr virus (EBV)-positive [5]. The treatment of HL-RT is based on the treatment of de novo classic Hodgkin lymphoma (cHL), which usually means the doxorubicin, bleomycin, vinblastine, dacarbazine (ABVD) regimen. HL-RT patients generally respond well to cHL therapy [6]. In the following, we present a case of HL-RT in an unexpected extranodal location, successfully treated with six cycles of brentuximab+doxorubicin, vinblastine, and dacarbazine (A+AVD) at our center.

## Case presentation

A 74-year-old woman with a history of hypertension and hyperthyroidism had been under observation in the Department of Internal Medicine, Division of Hematology, at the University of Debrecen since 2018 due to mild lymphocytosis (lymphocyte count was 5.4 G/L).

In 2019, flow cytometry of peripheral blood revealed 36% of B-CLL cells. Immunochemistry also confirmed B cell phenotype with CD19+, 20+, 23+, 43+, ROR+, CD5+, FMC7dim+, CD81-, CD38+ 25% status. She was diagnosed with CLL/SLL (stage 0 according to Rai classification). Fluorescence in situ hybridization (FISH) revealed a 13q deletion. Molecular analysis showed IGHV mutated status and TP53 mutation negativity. Unfortunately, we did not examine genetic lesions involving NOTCH1 and MYC activation. Histology of lymph node biopsy did not confirm malignancy, but the cervical lymph node fine-needle aspiration biopsy (FNAB) confirmed the diagnosis. We opted for a watch-and-wait approach.

By 2021, the patient developed B symptoms (weight loss, fatigue, and night sweats), with increased CRP level, high erythrocyte sedimentation rate (ESR) (100 mm/h), and grade 1 anemia. In April 2022, we started her treatment under a clinical trial protocol. Before therapy, a bone marrow biopsy showed normal hemopoiesis. Laboratory parameters are shown in Table 1.

**Table 1:** Laboratory parameters WBC: white blood cell; Hgb: hemoglobin; PLT: platelet; LDH: lactate dehydrogenase; CRP: C-reactive protein

	13/04/2022	15/06/2022	09/01/2024.	Normal values
WBC (G/l)	9.55	9.75	9.9	5.5-10.8
Lymphocyte (G/l)	4.34	2.82	1.8	0.9-3.1
Hgb (g/dl)	10.1	9.3	11.2	11.5-15.0
PLT (G/l)	310	263	322	150-400
LDH (U/l)	194	151	156	135-220
CRP (mg/dl)	31	36.2	22.8	<4.6

**Figure 1:**
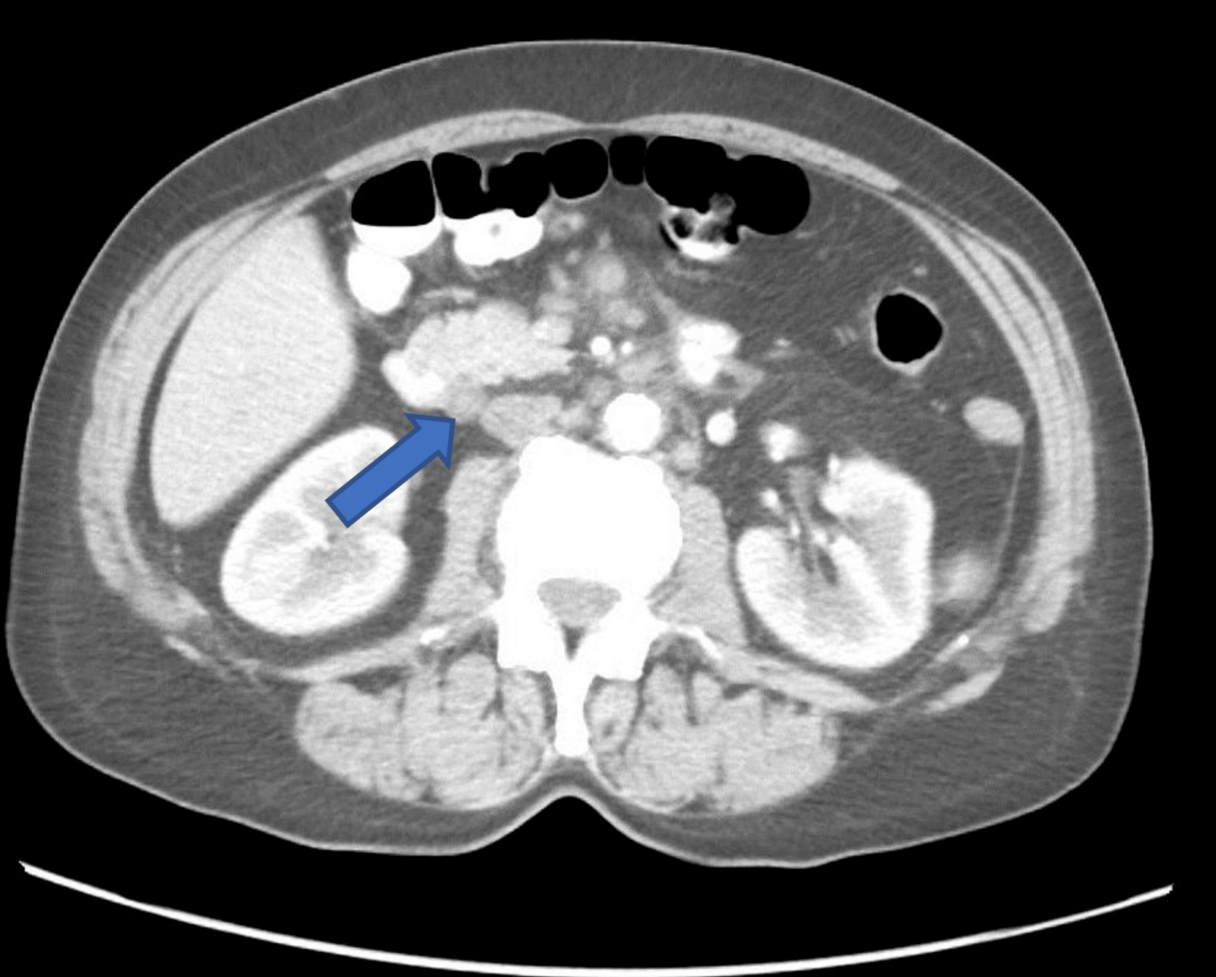
Abdominal axial CT showing enlarged paraaortic lymph nodes (arrow)

Hepatitis serologies (hepatitis B surface antigen and anti-hepatitis C virus antibody) were negative; toxoplasma, EBV, and cytomegalovirus (CMV) serologies showed transmitted infection (IgG antibodies were positive, but IgM antibodies were negative). Computed tomography (CT) imaging of the neck, chest, and abdomen revealed hepatosplenomegaly and generalized lymphadenomegaly (cervical, mediastinal, peritoneal, paraaortic, and parailiacal) (Figure 1).

The patient was started on a Bruton’s tyrosine kinase (BTK) inhibitor therapy and completed two cycles, resulting in partial remission. The laboratory tests showed grade 1 anemia and still high ESR. However, in June 2022, it was discontinued at the patient’s request, in part due to rheumatologic symptoms. Laboratory parameters at this time are shown in Table 1. 

Despite treatment discontinuation, rheumatologic complaints persisted, and she had rib and hip pain. Therefore, we further investigated her from a rheumatological aspect. During her observation, polymyalgia rheumatica (PMR) was confirmed, and she was started on a low-dose corticosteroid (methylprednisolone 8-6-4 mg) and sulfasalazine treatment, but the symptoms did not improve, although a mild improvement was observed in ESR (60 mm/h). Serum electrophoresis revealed monoclonal gammopathy (IgG kappa 3,7 g/l); osteolytic lesions were not detected. 

**Figure 2:**
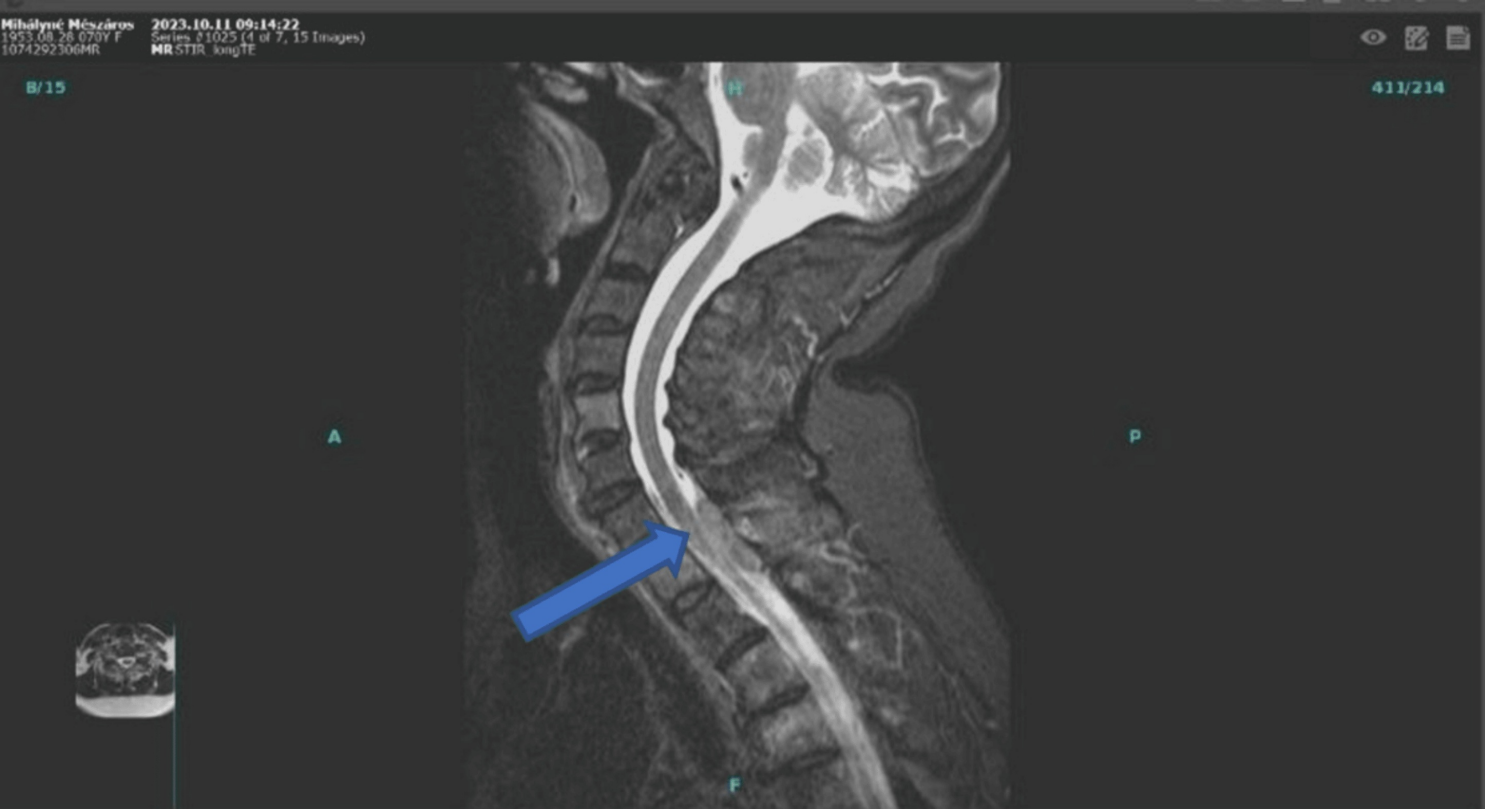
MRI showing intra- and extramedullary mass extending from cervical vertebrae (C1-C7) and the first thoracic vertebra (Th1) (arrow)

In August 2023, the patient developed severe cervicobrachialgia, which progressed to intolerable levels by October, accompanied by right arm numbness. CT and magnetic resonance imaging (MRI) revealed an intra- and extramedullary mass extending from cervical vertebrae (C1-C7) and the first thoracic vertebra (Th1) (Figure 2).

**Figure 3:**
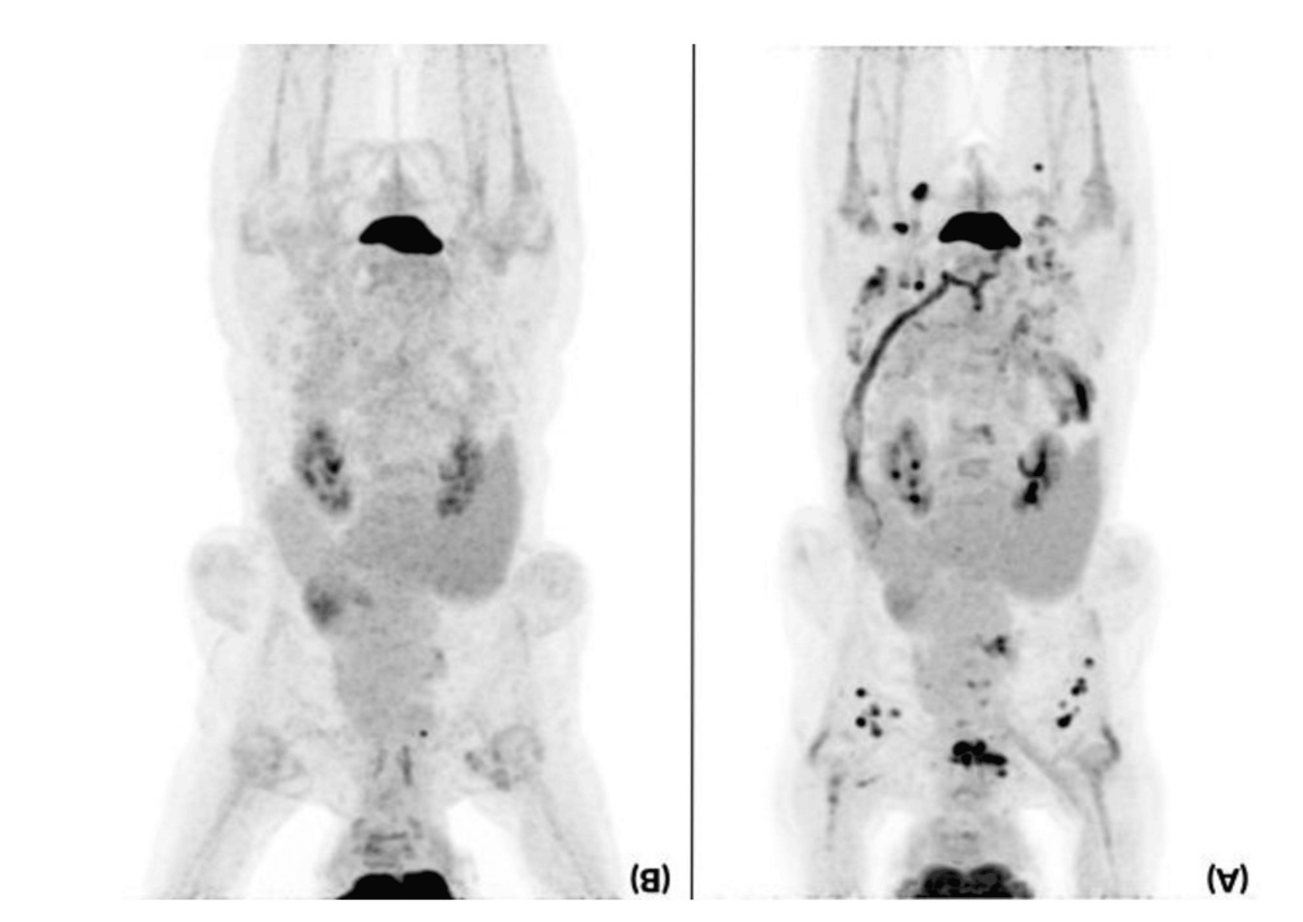
Full-body 3D PET images PET: positron emission tomography; CLL: chronic lymphocytic leukemia; A+AVD: brentuximab+doxorubicin, vinblastine, dacarbazine; CMR: complete metabolic remission; DS2: Deauville score; EOT: end of treatment (A) Supra- and infra-diaphragmatic enlarged hypermetabolic lymph nodes with bone involvement, including vertebral (C7-Th1) hypermetabolic lesions (SUVmax 15,3: standard uptake value), suggesting high-grade transformation of CLL (December 2023). (B) Following six cycles of A+AVD therapy, CMR DS2 was achieved on EOT imaging (August 2024)

In November 2023, surgical decompression and tissue sampling were performed; histology was inconclusive. Abdominal ultrasound detected a right parailiacal lymph node conglomerate, and the patient was complaining about abdominal pain. The molecular analysis of the tissue from the spinal cord did not support lymphoproliferative disease. The patients' general symptoms continue to worsen, hematochezia occurred, and neurological symptoms deteriorated; thus, a repeated MRI was performed, showing rapid progression of the tumor. Colonoscopy and histological examination of the colon revealed ulcerative colitis; thus, mesalazine was started. Due to persistent complaints, 18F-fluorodeoxyglucose positron emission tomography-computed tomography (18F-FDG PET-CT) was performed in December 2023, revealing maximum standard uptake value (SUVmax) enrichment at the C7 and Th1 levels, with disseminated bone and lymph node involvement and bone marrow hypermetabolism. The highest SUVmax was 15.3. Based on the image and progression, a possible RT was suspected (Figure 3A).

**Figure 4:**
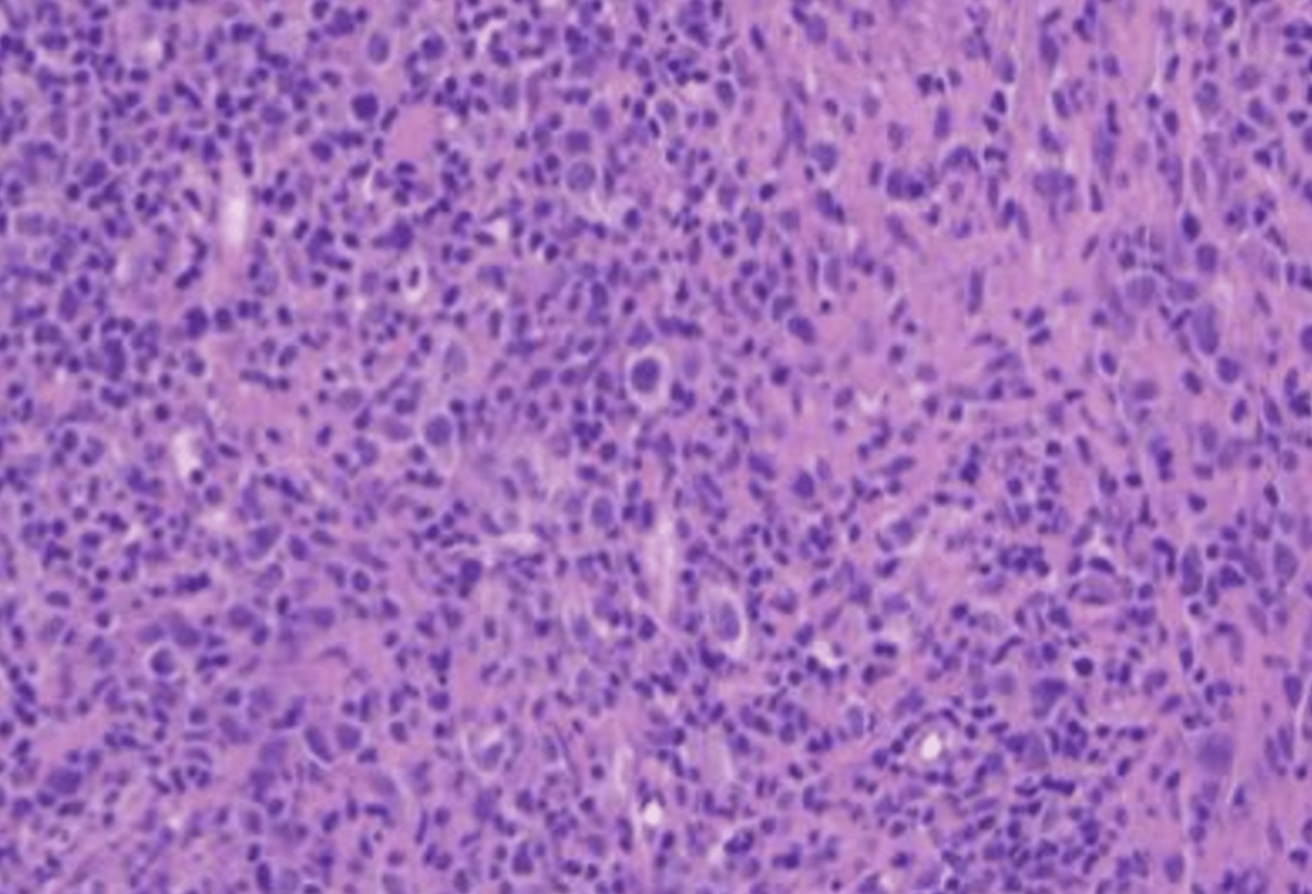
Lymph node core biopsy RS: Reed-Sternberg; HE: hematoxylin and eosin The basic architecture is partially preserved. Three ill-defined nodular structures can be identified within the sample, each containing scattered large atypical cells. These cells display large, occasionally lobulated nuclei, prominent nucleoli, and relatively broad, pale eosinophilic cytoplasm. Classic RS cells are not observed (HE stain, 20x)

In January 2024, a repeated lymph node core biopsy from an axillary lymph node confirmed HL-RT, with an International Prognostic Score (IPS) of 2, Ann Arbor stage of IV-B, and histology labelled as nodular sclerosis (NS) type (Figure 4).

**Figure 5:**
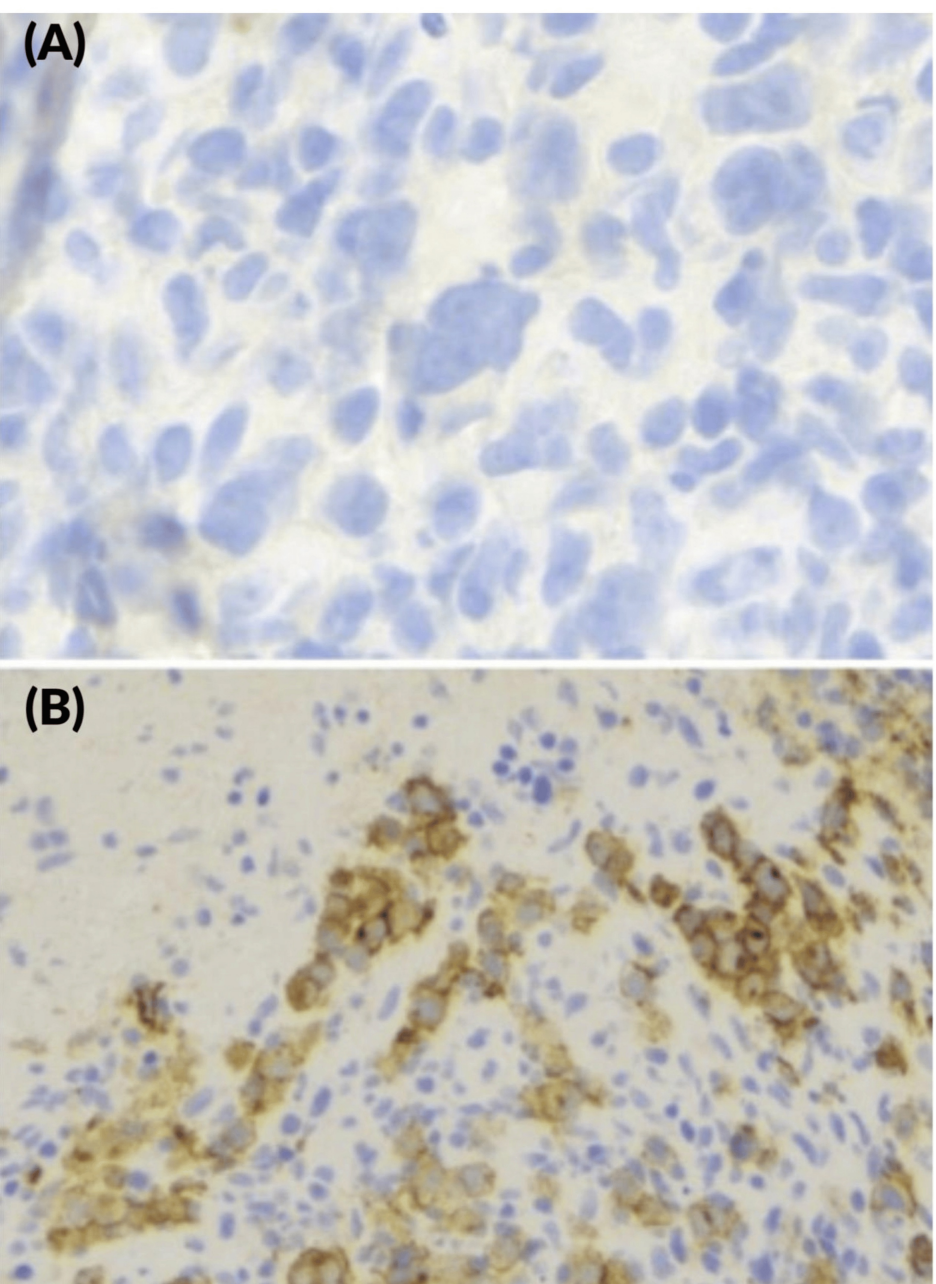
Lymph node core biopsy. Immunochemistry showing atypical cells negative for CD20 ((A) x40) and positive for CD30 ((B) x20)

The immunophenotype expression pattern was as follows: CD30+, PAX5+, MUM1+, LCA-, CD79a+/-, CD20-, CD138-, ALK-, CD5-, CD23-, LEF1 +/-, CD15 +/-, BCL6-, CD3-, and LMP1- (Figure 5).

**Figure 6:**
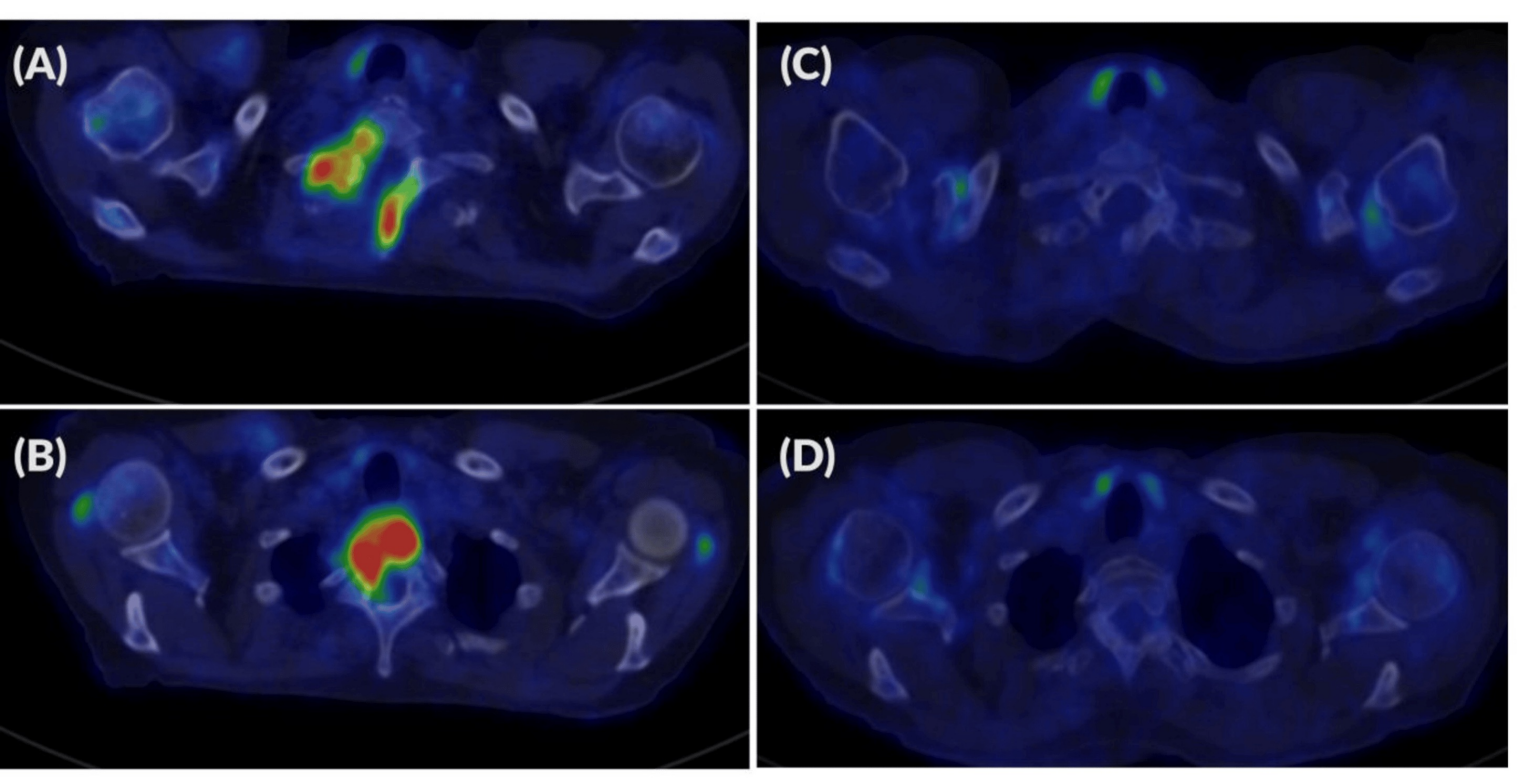
Axial PET-CT images PET: positron emission tomography; CT: computed tomography; CLL: chronic lymphocytic leukemia; A+AVD: brentuximab+doxorubicin, vinblastine, dacarbazine; CMR: complete metabolic remission; DS2: Deauville score; EOT: end of treatment Hypermetabolic lesions with SUVmax of 14.5 were observed along the laminae of the C7 vertebra (B) and in the vertebral body of Th1 (A), postoperative state following spinal decompression surgery (December 2023). (C), (D) Following six cycles of A+AVD therapy, CMR DS2 was achieved on EOT PET-CT (August 2024)

**Figure 7:**
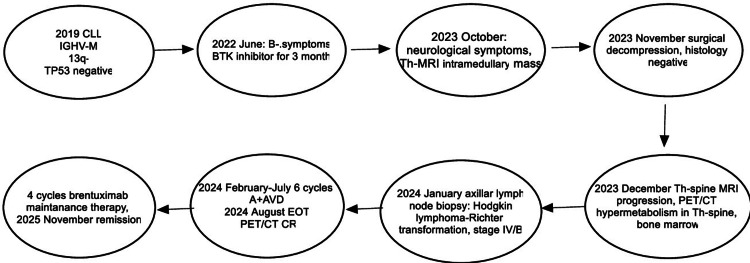
Flow chart of the case report CLL: chronic lymphocytic leukemia; IGHV-M: immunoglobulin heavy chain mutated; Th-MRI: thoracic spinal magnetic resonance imaging; A+AVD: brentuximab-doxorubicin, vinblastine, and dacarbazine; EOT: end of treatment; CR: complete remission; PET: positron emission tomography

At that time, peripheral blood counts are shown in Table 1. The patient received six cycles of A+ AVD (brentuximab vedotin 50 mg, doxorubicin 40 mg, vinblastine 9 mg, dacarbazine 600 mg) under close cardio-oncological monitoring due to confirmed aortic stenosis (aortic valve area (AVA): 0.9 cm2). As supportive treatment, she received antiemetic and gastric protective treatment, colony-stimulating factor (filgrastim), and antiviral (acyclovir) and antibacterial prophylaxis (co-trimoxazole). After the first cycle of treatment, she developed neurological symptoms, urgent brain CT and MRI excluded any ischemia or intracranial bleeding, but we observed severe grade 4 hyponatremia (as a chemotherapy-related side effect). By using slow sodium replacement, her symptoms improved. After this side of the intervention, she received four more cycles of brentuximab (50 mg) as maintenance therapy. In August 2024, end-of-treatment (EOT) PET-CT showed CMR and a Deauville score of 2 (Figures 3B, 6C-6D) [7]. After the EOT PET/CT, she underwent successful transcatheter aortic valve implantation (TAVI) in October 2024. After the intervention, she received four more cycles of brentuximab vedotin (50 mg) as maintenance therapy. The flow chart of the case is shown in Figure 7. A cardiological check-up in April 2025 confirmed good clinical status. Hematologic check-ups every three months showed no progression (last control was done in November 2025).

## Discussion

CLL is the most common type of leukemia in adults. In most cases, the disease course is indolent, with prolonged survival even without treatment. Recent treatment options, including B-cell lymphoma 2 (BCL-2) inhibitors or BTK inhibitors, have revolutionized CLL management [8]. The risk of transformation to HL-RT is low, approximately 0.5% within 10 years of diagnosis [9]. However, RT remains a feared complication. RT is characterized by rapid clinical progression. The prognosis of HL-RT is generally unfavorable and may be fatal without timely intervention. While outcomes in HL-RT are typically worse than in de novo HL, they are more favorable compared to DLBCL-RT [10,11].

Identified risk factors for RT include advanced age, advanced disease stage, lymphadenopathy with nodes larger than 3 cm, unmutated IGHV, the presence of a 17p deletion and/or a TP53 mutation, NOTCH1 mutation, and a stereotyped B-cell receptor (BCR).

The role of EBV positivity as a predisposing factor in RT has been under investigation for a long time, particularly in clonally unrelated cases of HL-RT [6,12]. An American study from 2016 demonstrated that RS cells were EBV-positive in 71% of HL-RT patients. Most studies report similar findings, with EBV positivity observed in 67-75% of HL-RT cases, further supporting its possible pathogenic role [13].

Therapeutic management of HL-RT usually follows the same approach as for de novo cHL, typically involving the ABVD regimen. In a 2024 American study, four patients with HL-RT were treated with the A+AVD regimen. Two of them achieved successful outcomes, with durable remission lasting 40 and 42 months, respectively, while the other two experienced disease progression and subsequently died [8]. Although evidence is limited, these results indicate that A+AVD can be effective in some HL-RT cases. Our patient responded well, achieving complete remission after six cycles of A+AVD. Spinal cord involvement has been well-documented in cases of cHL, and there are also reports of central nervous system (CNS) involvement in RT [14]. However, these CNS manifestations have exclusively been described in the DLBCL variant of RT [15]. The diagnosis of RT in our patients was difficult; it took more than six months to establish the correct diagnosis. The cytogenetic abnormality (13q deletion) and the IGHV-M status are known to be good prognostic factors in CLL. In our case, the development of RT was unlikely; moreover, unless there were B-symptoms and a high sedimentation rate, there were no other serious abnormalities that would have suggested such a complication. To our knowledge, no prior case of spinal cord involvement in HL-RT has been reported in the literature, making this case unique and novel.

## Conclusions

In conclusion, we presented the case of a CLL patient, where HL-type RT manifested in an unusual anatomical location, the spinal cord, which is rarely described in the literature. This highlights the importance of considering RT even in atypical extranodal presentations, especially when accompanied by neurological symptoms. Furthermore, the absence of classical molecular risk factors such as TP53 mutation, unmutated IGHV status, or the lack of 13q deletion emphasizes that HL-RT may arise even in seemingly low-risk CLL patients. The patient responded well to A+AVD chemotherapy, achieving CMR, which suggests that brentuximab-containing regimens may offer effective therapeutic options in HL-RT, even in elderly patients with comorbidities such as aortic stenosis. This case underlines the need for individualized therapeutic strategies and vigilant long-term monitoring in CLL patients, as RT can occur unpredictably and may present with misleading or nonspecific clinical features. Continued reporting of HL-RT cases is essential to improving understanding and guiding optimal management of this rare and heterogeneous condition. Specific risk factors for the development of HL-RT have yet to be identified.
